# Retraction: Innate Immune Sensing of Modified Vaccinia Virus Ankara (MVA) Is Mediated by TLR2-TLR6, MDA-5 and the NALP3 Inflammasome

**DOI:** 10.1371/journal.ppat.1010263

**Published:** 2022-01-25

**Authors:** The PLOS Pathogens Editors

After this article [1] was published, the corresponding author contacted the journal to notify the editors of image duplication concerns affecting Figs 6, 9, and S1.

Specifically, the following concerns were raised:

- In Fig 6, panel B, the band in lane 3 of the IPS-1 blot appears similar to the band in lane 3 of the MDA-5 blot of S1 Fig, panel D, when rotated.
- In Fig 9, panel A, the total ERK1/2 blot appears similar to lanes 3–7 of an unlabeled 9-lane blot found in an earlier unpublished reference document. In S1 Fig, Panel F, lanes 2–4 of the P-ERK1/2 blot (MVA stimulation) appear similar to lanes 7–9 of the unlabeled 9-lane blot found in an earlier unpublished reference document. The band shown in lane 5 (24 h) of the total ERK 1/2 blot in Fig 9, panel A appears similar to the band shown in lane 2 (1 h) of S1 Fig, panel F, P-ERK12 blot, when flipped vertically.
- In Fig 9, panel A, the total JNK blot appears similar to the total STAT-1 blot of an earlier unpublished reference document.
- In S1 Fig, panel D, the tubulin blot appears similar to the last 5 lanes of the tubulin blot of panel F, flipped horizontally.

The corresponding author requested retraction of this article because the above image issues raise concerns about the scientific accuracy of several figures of the article.

Editorial assessment identified an additional concern that the pro-IL-1β panel in Fig 8D appears similar to lanes 7–10 of the tubulin panel in S6 Fig when re-sized vertically.

The corresponding author stated that the original autoradiographs underlying the above-mentioned figure panels are not available.

In light of the above concerns and in accordance with the request of the corresponding author, the *PLOS Pathogens* Editors retract this article.

TR, DLR, VP, CEG, BP, GP, ME and TC agreed with retraction. JD disagreed with retraction. QGST, MKR and SA either could not be reached or did not respond directly. JT is deceased. CEG, BP, and ME stand by the article’s findings. TR, DLR, VP, and TC apologize for the issues with the published article.
